# Unraveling the Cretaceous-Paleogene boundary event across the Gulf of Mexico—High-resolution Rayon reef section, Valles-San Luis Potosi platform, Mexico

**DOI:** 10.1371/journal.pone.0345692

**Published:** 2026-04-22

**Authors:** Roberto Bartali, Jaime Urrutia-Fucugauchi, Jose Ramon Torres-Hernandez, Ligia Perez-Cruz, Rosa Lina Tovar-Tovar

**Affiliations:** 1 Facultad de Ciencias, Universidad Autónoma de San Luis Potosi, San Luis Potosi, México; 2 Programa Universitario de Perforaciones en Oceanos y Continentes, Instituto de Geofisica, Universidad Nacional Autonoma de Mexico, Mexico, Mexico; 3 Instituto de Investigación Científica y Estudios Avanzados Chicxulub, Parque Científico Tecnológico de Yucatán, Sierra Papacal, Merida, Yucatán, Mexico; 4 Instituto de Geología, Universidad Autónoma de San Luis Potosi, San Luis Potosi, Mexico; 5 Coordinación de la Investigación Científica, Universidad Nacional Autónoma de Mexico, Mexico, México; 6 Instituto de Metalurgia, Universidad Autónoma de San Luis Potosi, San Luis Potosi, Mexico; Hefei University of Technology School of Resources and Environmental Engineering, CHINA

## Abstract

The Cretaceous/Paleogene boundary, marked by the Chicxulub impact ejecta layer has a global distribution, with distal, intermediate and proximal boundary sections of distinct mineralogy, geochemistry and stratigraphy. Distal sections show a thin double-layer of spherulites and clay, enriched in platinum group elements and impact minerals. Proximal boundary sections around the Gulf of Mexico-Caribbean Sea are thicker, with clastic tsunami and debris flow deposits and the fireball layer in between the spherulitic and clay layers. Impact-induced disturbance and stratigraphic gaps have limited the resolution in unveiling the event sequence. Here we present a study on a recently discovered reef section in a carbonate platform, ~ 1,050 km away across the Gulf of Mexico. The boundary is marked by a black clay with shock quartz, glassy spherules, metallic particles, broken zircons, gypsum crystals with fragmented spherules and platinum group elements (Ir, Os, Pd). Fossils in the black clay are absent and only fossil evidence at the bottom is charred matter, covered by lignite mixed with spherules. Corals and pelecypod shells are incrusted by spherules. The lower unit is formed by cream-colored sandstones of a rudist-coral reef environment with variable thickness and the upper unit by sandstones with few gastropods and fragmented shells. The Rayon reef in the Valles-San Luis platform provides a high-resolution proximal record of Chicxulub impact in the Gulf of Mexico.

## Introduction

The End-Cretaceous mass extinction at the Cretaceous/Paleogene (K/Pg) boundary is associated to climatic and environmental effects of an asteroid impact [1,2]. The globally distributed impact layer is marked by the iridium anomaly, initially documented at K/Pg boundary sites in Italy, Denmark and New Zealand [1]. Distal boundary sites are characterized by the spherule-clay double layer, enriched in platinum group elements (PGE) as well as shock quartz and impact minerals with high pressure planar deformation (PDF) features [1,2]. Geochemical and mineralogical evidence for the K/Pg impact continues being unraveled from sites in marine and continental sections worldwide [2].

The Chicxulub crater formed by an asteroid impact ~66 Ma ago on the Yucatan carbonate platform in the Gulf of Mexico [3]. Impact-forming craters are highly energetic events. Cratering involves deep transient excavation cavities, basement uplift, faulting and ejection/emplacement of breccias and impact melt, accompanied by plume collapse, block displacement, secondary cratering and high-temperature lateral curtains and ground surges. The impact-induced deformation and ejecta and mass transport, with debris flows and tsunami resulted in widespread impact and debris deposits in the areas surrounding the impact site.

Boundary sections at varying distances from impact site provide records of impact dynamics, ejecta, impact-induced deformation, climate change and event sequence. Around the Gulf of Mexico-Caribbean Sea, sections show increased thickness and more complex stratigraphy, characterized by tsunami, debris flows and hiatuses [4–7]. The study site in the Rayon reef is located in the Valles-San Luis carbonate platform, which is located to the west of the Gulf of Mexico coastal plain. Rudist-coral and coral-dominated reefs develop in the extensive carbonate platform, which provided a relatively protected area during the impact-induced deformation and tsunami and debris flows triggered by the Chicxulub impact in the Yucatan platform.

In the Gulf of Mexico, Chicxulub ejecta deposits show a characteristic basal spherulitic layer covered by the thick massive clastic deposits, which record target deformation, cratering and margin collapse triggered by high-energy release of the impact and cratering. The clastic unit is covered by the fireball layer resulting from the reentry of ejecta into the upper atmosphere and the clay layer. Fireball is here used for the impact-induced high-temperature release and the thermal pulse generated by massive ejecta reentry to the atmosphere. The clay layer formed by the globally distributed thin ejecta deposited over an extended period, blocked solar radiation and photosynthesis and triggering the extinction event [1,2].

**Fig 1:**
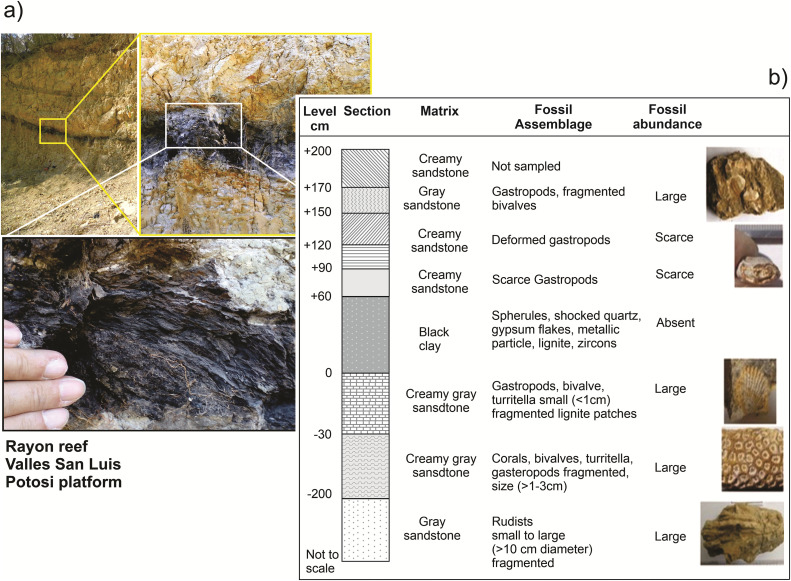
Rayon reef section, Valles-San Luis Potosi platform, northeastern Mexico. (a) Rayon reef outcrop images, with the black clay layer that marks the K/Pg boundary. (b) Schematic section column, with descriptions of matrix, fossil assemblage, abundance and fossil images.

We present the results of a study of the Rayon reef K/Pg boundary section in the Valles-San Luis Potosi platform across the Gulf of Mexico, ~ 1,050 km away from impact site (Fig 1). The recently discovered Rayon reef section preserves a detailed record of the Chicxulub impact in a proximal section, in contrast to circum-Gulf of Mexico sections in northeastern Mexico, which high-energy clastic deposits, reworking and hiatus limit their stratigraphic resolution.

### K/Pg boundary

K/Pg boundary sites show a global distribution, with distal, intermediate and proximal sites providing information and constraints on ejecta distribution and impact-induced effects. The boundary layer is characterized by anomalous contents of iridium and platinum group elements, shocked quartz, magnesioferrites and impact markers [1,2,8]. In the Gulf of Mexico and Caribbean Sea, the boundary sections include a basal spherulitic-clay layer and in between, thick clastic deposits and the disturbance and thermal effects associated with crater formation, tsunami and the ejecta reentry in the upper atmosphere. Studies provide increasing resolution records of the impact and K/Pg boundary events [e.g., 9–14]. Nevertheless, stratigraphic and fossil record incompleteness limit the temporal resolution for resolving the event sequence [15].

Chicxulub crater formed on an extensive carbonate platform built on Yucatan Block crystalline-metamorphic basement [16–18]. The impact excavated a deep transient cavity, with deformation and fragmentation of crustal materials forming an impact plume and lateral curtains [14,18]. The ejecta, characterized by mixtures of carbonate, basement and bolide components, reached a global distribution [1,2,19]. The crater was covered by carbonate sediments, which have preserved the structure and ejecta and at the same time present challenges for crater studies and reconstruction of the event sequence.

Boundary sections have been reported for northern and southern Mexico locations [20–25]. The nearest proximal sections are in the Yucatan peninsula, in southern Quintana Roo and central Belize [26]. The impact sections have been also investigated in drilling programs, within the crater and nearby localities [27–30]. High-resolution K/Pg boundary records include the Blake Nose drill sites, which preserve the proximal impact ejecta and fireball layer in between the Maastrichtian and Paleocene carbonates [11]. An intermediate distance high-resolution record to the south is the Gorgonilla island boundary section, Colombia, which preserves a spherule-rich bed within a turbiditic sequence of litharenites and tuffaceous marls [31]. Proximal Gulf sections as mentioned above show in general complex stratigraphy, affected by tsunamis, slumping and debris flows that limit their temporal resolution and stratigraphic completeness. Studies on high-resolution impact records are needed.

DePalma et al [32] presented results of a study of Tanis site in the Hell Creek Formation, North American interior seaway, which is associated with a seismically induced surge in a fluvial marine onshore setting. The deposit shows evidence of rapid emplacement under turbulent conditions, with a mixed chaotic fossil assemblage that contains melt glassy spherules, microkrystites, shocked minerals with planar deformation, unaltered impact melt glass, and an iridium enriched fine grained ejecta cap. The ~ 1.3 m thick graded clastic unit underlying the cap iridium enriched tonstein and fossil assemblage record the impact effects, ejecta emplacement and local disturbance and depositional processes.

## Rayon reef boundary section

The study area in the Valles-San Luis Potosi platform lies farther to the east and south of boundary sections in northeastern Mexico [33]. Paleoreconstructions of the circum-Gulf of Mexico show complex relief associated to thrusting and folding during the Laramide orogeny in Late Cretaceous and Paleogene.

The Rayon reef is characterized by a Late Cretaceous shallow platform environment. The section in the Cardenas Formation is formed by thick clastic-carbonate sediments overlying conformably carbonate sediments of the Valles-San Luis Potosi platform. Myers [34] described the Cardenas Formation formed by three members of marls, silts and sandstones, with the basal and upper members characterized by coral-rudist limestones. Studies have investigated the stratigraphy, depositional environment and fossil contents of the Cardenas Formation, as well as the structural and tectonic deformation in the Sierra Madre Oriental [34–38]. In the section, the most abundant fossil assemblage corresponds to rudists and corals. The overlying upper sandstone layers contain isolated small specimens of rudists, corals and abundant turritellas.

The abundant rudists and scleractinian corals are well preserved and have been described in several studies [35–38]. Coral species described in the rudist-coral and coral assemblages include sixteen taxa from nine families, with *Siderastrea adkinsi, Actinhelia elegans, Cladocora gracilis, Dictuophyllia conferticostata, Multicolumnastraea cya thiformis, Dermosmiliopsis orbignyi, Placocoenia major, Actinacis haueri and Acti nacis parvistella* [35]. The rudist assemblage documented in Pons et al [37,38] include the radiolitids *Huasteca ojanchalensis* (Myers), *Biradiolites aguilerae* Böse, *B., muellerriedi* (Vermunt), *Cárdenasensis* Böse, *Trechmannites rudissiums* (Trechmann), Praebarretia sparcilirata (Whitfield), hippuritids *Caribbea Tampsia floriformis* (Myers), plagioptychids *Coralliochama gbohemi* Böse and *Mitrocaprina tschoppi* (Palmer). Rudists of genera *Huasteca* and *Trechmannites* were also observed [38].

Rudists were abundant in the benthic carbonate environments of Tethys seaway [37,38], which went extinct at the end of the Cretaceous. The gastropods are widespread in the Huasteca Potosina, especially near the town of Cardenas 20 km away to the north. The Campanian-Maastrichtian Cardenas Formation is equivalent to the deep-water marls of Mendez Formation to the east in the Gulf coast foreland basin. The Cardenas Formation is on top of the shallow water carbonates of the Santonian Tamasopo Formation and overlain by the Tabaco Formation [36–38].

## Methods

### Sampling

The Rayon reef outcrop section studied is approximately 12 m long and 6 m high and shows distinct lithology and fossil contents. The K/Pg boundary marked by the black clay horizon is overlying/overlain by sandstone layers (Fig 1). Initial analyses focused on outcrop description of the stratigraphy, lithology and fossil contents. The black clay shows distinctive mineralogic and chemical composition, absence of fossil remains and presence of impact indicators. The clay shows a compact high-density appearance that disintegrates with relative ease. It is well preserved, although some parts are eroded due to the fragility of the clay, particularly in the lower layers that are the most heterogeneous.

The section was sampled, with samples collected every 5 cm from 1 m below to 1 m above the black clay that was used as the reference level. Samples were additionally taken from side profiles at about 2 m to the right and to the left. Samples collected up to 20 cm below the reference line at the base of the black clay and up to 20 cm above the black clay were used for laboratory analyses. Subsamples were characterized and analyzed on petrographic microscope, scanning electron microscope and X-ray diffraction.

### Laboratory analyses

Samples were cut into subsamples and analyzed on a petrographic microscope, scanning electron microscope SEM, energy dispersive spectroscopy EDS and X-ray diffractometer (XRD). Gypsum and calcite particles were further analyzed by Raman and Fourier Transform Infrared Spectroscopy (FTIR). Analyses aimed to constrain the inner structure and variations in elemental content. Analyses document quartz grains with impact deformations, spherules of various types, lignite and metal particles, in addition to tabular gypsum crystals that present characteristic high pressure deformation features. The black clay shows a compact high-density appearance; however, it has a fine granular poorly consolidated structure, with particle sizes from 1 to 100 μm, which can be broken up by applying pressure. This facilitated separation of lignite, spherules, crystals and metallic particles, without need for crushing or melting samples, preserving the fragile gypsum crystals and spherules. The upper and lower cream-colored sandstone units, despite showing granular structures with large grain sizes, are more resistant to disaggregation. Samples were crushed using a lightweight hammer.

Petrographic observations were made with Zeiss and Olympus petrographic microscopes. SEM and EDS analyses were carried on a JEOL scanning electron microscope model JSM-6610LV. X-ray diffraction analyses were made with the Bruker model D8 Advanced X-ray diffractometer. The Brucker D8 Advance X-ray difractometer is equipped with a LYNXEYE detector, with twin-twin optics and automatized sample holder. The analysis was carried out with the Difract.eva software 5.1 with the data base PDF-2 ICDD Release 2022 and TOPAS 4.2 software.

Raman analyses to characterize the crystals, gypsum flakes and metallic particles were made in a Raman spectrometer real-time performance target instrument GW INSTEK LCR-819 and a Horiba Raman spectroscope Xplora Plus with 532 nm laser line. Analyses were also carried out with Brucker Fourier Transform Infrared Spectroscopy FTIR spectroscope Vertex 70 Hyperion with a range of 380 to 25.000 cm^-1^ and a NT-MDT atomic force microscope AFM Solver Next.

Iridescence was examined using an optical microscope illuminating the sample at different angles with a variable brightness white LED light.

The metallic particles were separated using a magnet. Powder was distributed on white paper sheets and a neodymium magnet slid under to separate the magnetic particles. The magnet was wrapped by a layer of clean pack to prevent contamination. When possible, individual particles were collected with magnetic steel needles 1 mm in diameter and 5 cm long and attached to adhesive tape.

## Results

### Rayon boundary section

As mentioned in previous section, the K/Pg boundary section is marked by a black clay that shows absence of fossil remains and distinct mineralogical and chemical composition. The black clay, characterized by charred plant matter at the bottom, lies in between distinct sandstone units. The lower unit is formed by Late Cretaceous cream-colored reef sandstones of variable thickness, between 2 and 6 meters. The upper sandstone is characterized by occurrence of few gastropod fossils, as compared with the lower sandstone. The light grey silts located 2 m above the black clay contain gastropods of ~1-1.5 cm in diameter, while at the level immediately below the gastropods are mostly crushed with no traces of other fossil remains.

Unlike other outcrops in which fossils are mixed or in reverse stratification (e.g., rudists above the corals), fossils in the lower sandstones appear arranged in sequence depending on water depth in the reef. The sequence shows in ascending order layers of rudists, corals, pelecypods and gastropods (Fig 1). The size of gastropod and pelecypod shells decreases with position in the outcrop upwards to the black clay. The most abundant assemblage corresponds to rudists [34]. Rudist reefs were very abundant in the Cretaceous extensive carbonate platforms in the Americas, till they went extinct in the latest Cretaceous [39,40]. The overlying layers contain isolated small specimens of rudists (radiolitids), corals (no coral colonies) and abundant turritellas.

The corals are from several families and mixed with several species of turritellas. The gastropods near the coral-dominated zone reach 15 mm in diameter and up to 50 mm in length and are mixed with various types of pelecypods. The branches of corals, at the bottom of the outcrop, reach 20 mm in diameter, while those at the top are smaller, only about 5 mm in diameter. This suggests that water depth was decreasing and at the upper level was close to the beach.

In the outcrop, the base of the black clay is used as a reference level. The black clay is ~ 12 m in length and ~0.3 m thick (in its thickest part up to 0.6 m), oriented N30°W with an inclination of 25º towards the SW (disappearing below the road). The black clay shows a complex composition and layering, with three subunits distinguished. The basal subunit contains lignite and charred matter, with high contents of calcite and gypsum crystals and spherules of different types. The intermediate subunit is most complex. with two layers. The lower one, in addition to abundant spherules, presents lenticular crystals of gypsum with multiple layers of tabular crystals. The upper level contains metallic particles of different morphologies such as nuggets, spherules, branched and irregular. Particles are loose and others embedded in quartz and crystals, forming elongated aggregates. The top layer shows abundant gypsum with lenticular aggregates, crystals are scarce, with metallic particles embedded or in aggregates of small grains of sand. Lenticular gypsum crystals are only found in a stratum with an average thickness of 5 cm, not exceeding 10 cm. Tabular crystals are mostly in a stratum above.

The upper and lower sandstone units, despite being granular structures with larger grain sizes, are more resistant to disaggregate. The clastic sequence is a compact aggregate of fine particles from 1 to 100 μm, which facilitated sampling including fragile gypsum crystals. Dust particles identified under an optical microscope were scanned with a magnet to separate magnetic particles and analyzed by SEM. The black clay contains quartz grains with impact deformation, spherules of various types, lignite and metallic particles, in addition to tabular gypsum crystals with spherules, zircons, microtektites and shock deformation features.

**Fig 2:**
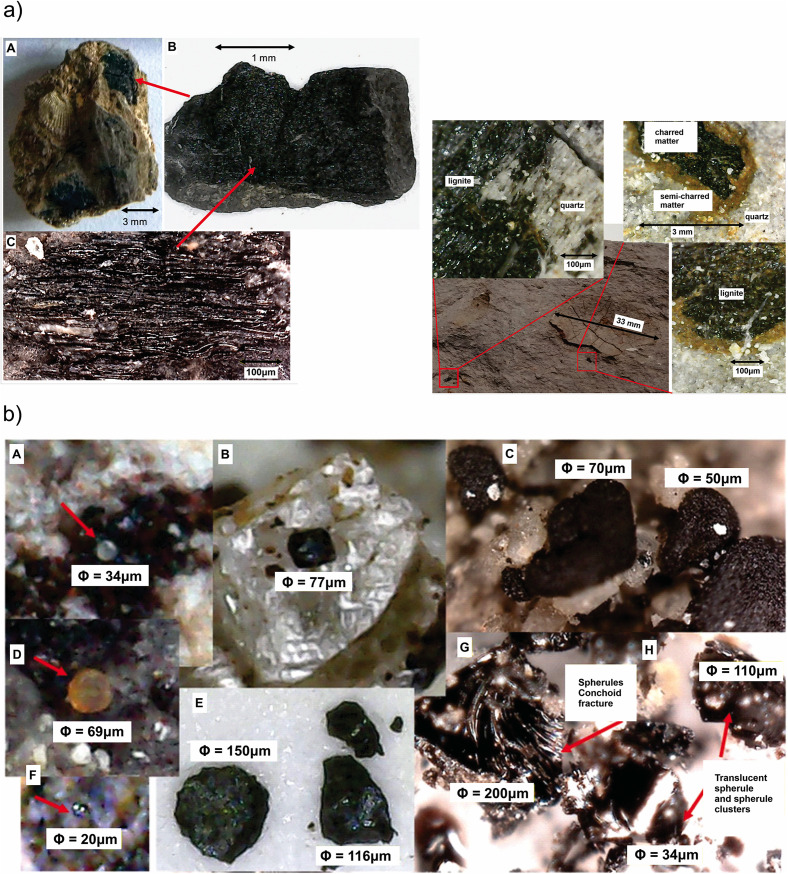
Rayon K/Pg boundary section with the diagnostic impact indicators (see text). (a) Lignite patches and carbonized vegetal matter fibers. Views of lignite and charred and semi-charred material. (b) Spherules, fragmented spherules, spherules with conchoid fractures, translucent spherules and spherule clusters, quartz, impact glasses, lignite, gypsum, metallic particles and nuggets in the black clay layer.

**Fig 3:**
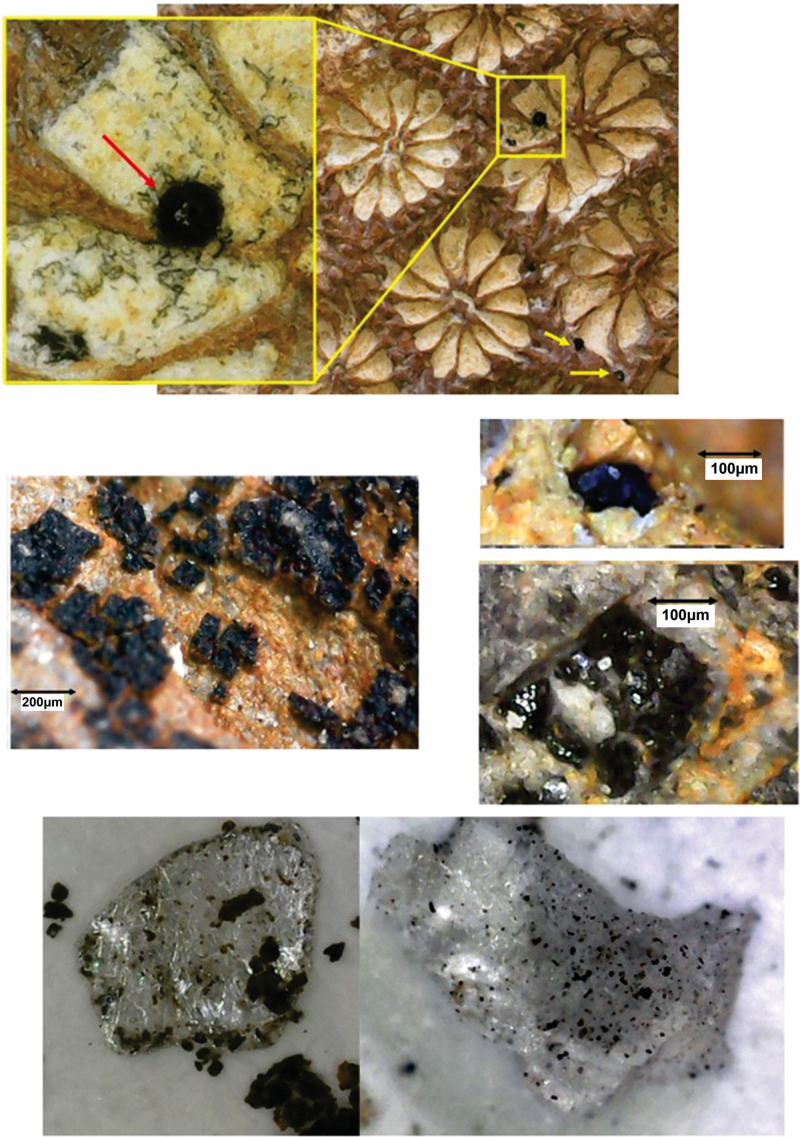
Spherules on fossil shells, withspherule incrusted on coral, spherules incrusted on shells and spherules in crystals.

**Fig 4:**
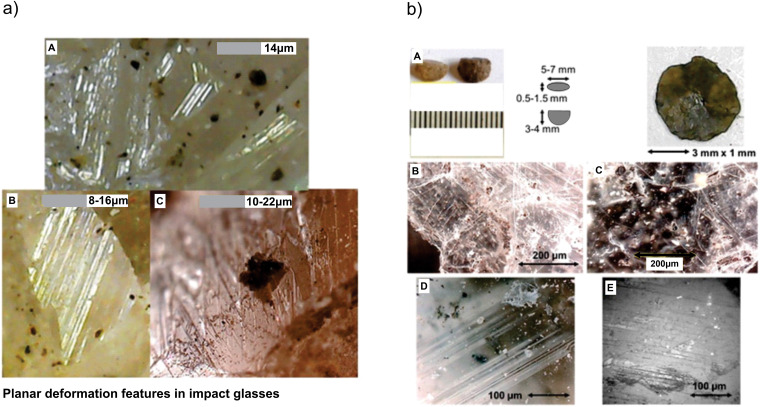
Quartz crystals showing distinct planar deformation features (PDFs). (a) Planar deformation features in impact glasses. (b) Views of planar deformation features, with different orientations (see text).

**Fig 5:**
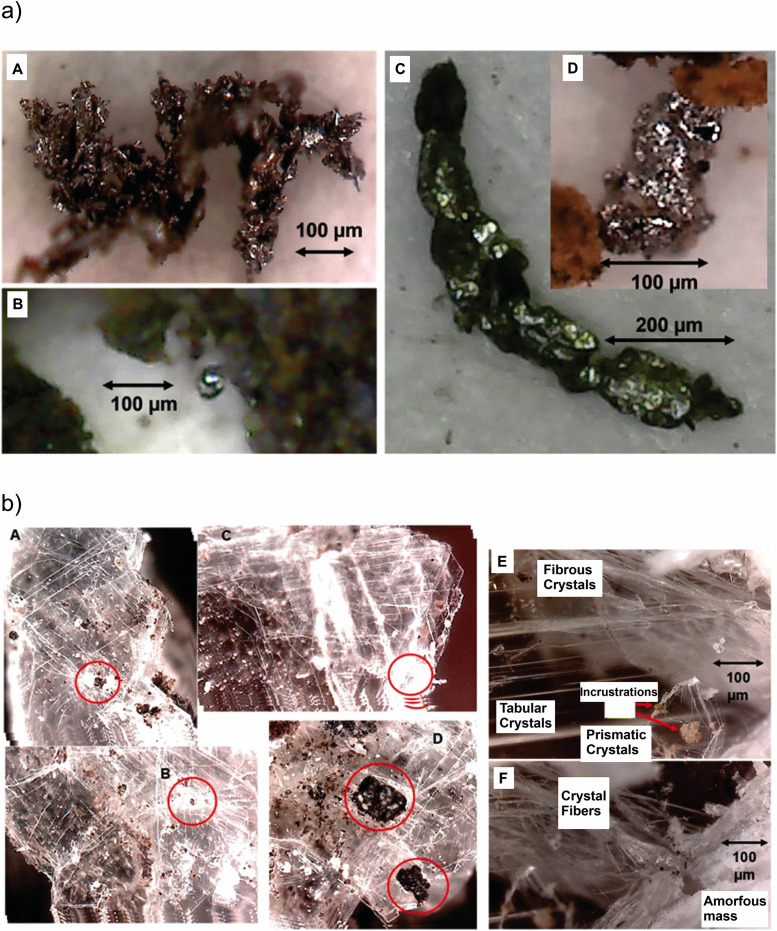
(a) Metallic particles and particle agglomerates, views of different sizes and geometries. (b) Gypsum tabular, prismatic and fibrous crystals, with planar deformation features.

**Fig 6:**
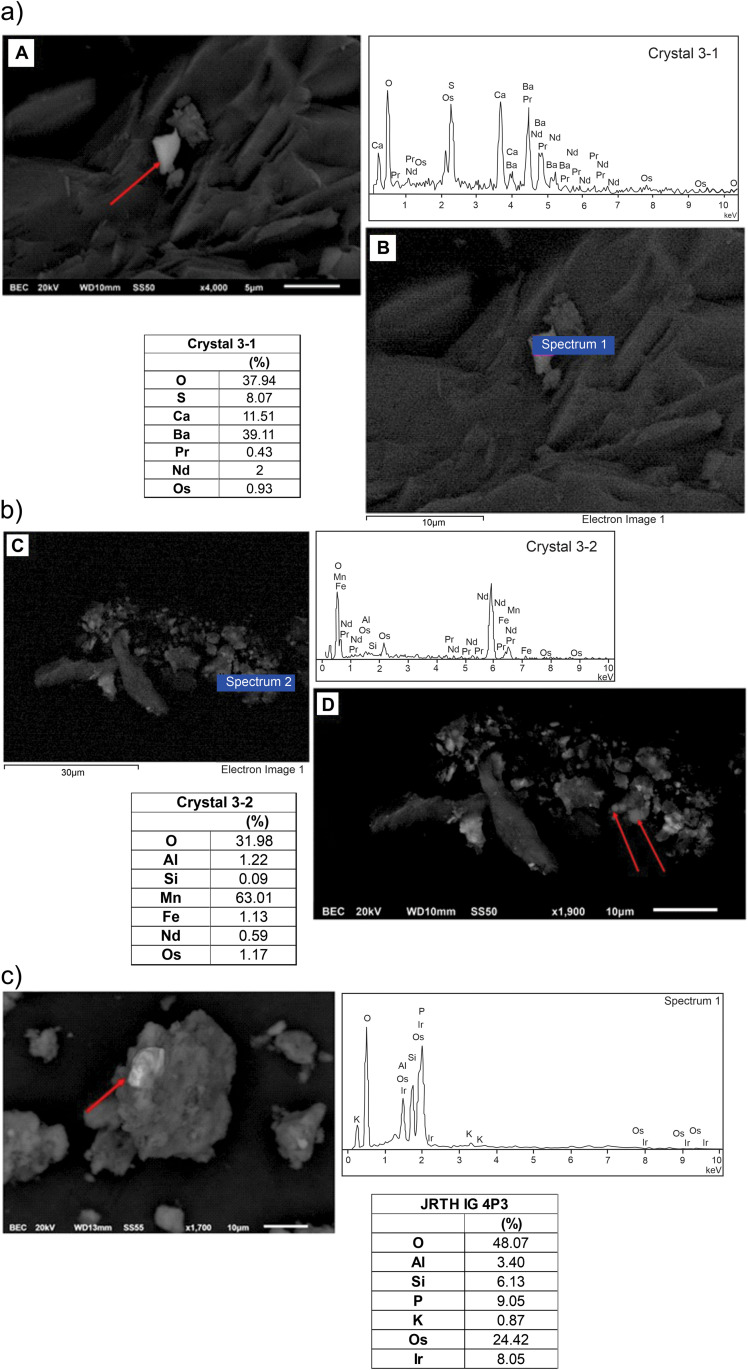
Scanning electron images and elemental analysis of crystals, with enrichment of PGE. (a) Crystal 3−1 with Pr, Nd and Os. (b) Crystal 3−2 with Nd and Os (c) Crystal JRTH IG 4P3 with Os and Ir.

The basal layer shows charred and semi-charred vegetation, with lignite patches (Fig 2a). The black clay is rich in spherules of different characteristics, including fragmented spherules (Fig 2b). Whole and fragmented spherules are incrusted on the corals and pelecypod shell remains (Fig 3). Quartz crystals show planar deformation features with distinctive patterns (Fig 4). The clay mineralogical composition differs from the sandstone units above and below, with characteristic gypsum and metallic particles (Fig 5). The basal contact shows distinctive features suggesting a sudden high energy event. Lignite, black spherules and quartz are observed in the thin layer of milky appearance, composed of white microcrystals (usually opaque and some of vitreous luster). It is common to find spherules of all kinds scattered among the crystals. Another characteristic of the black clay is gypsum in a thin layer 5–7 cm wide along the entire outcrop, with tabular crystals of gypsum spearheads and sizes ranging from less than 1–10 mm (Fig 6). The average value of the crystal´s major axis is 3–4 mm. They are bright and have vitreous luster, with reflection silver-yellowish and iridescence that highlight the fractures. There is abundant lignite, spherules and micro-fragments 5–50 μm of various minerals and abundant metallic particles.

### Spherules

Spherules are found at the base of the black clay next to abundant lignite-covered and charred matter (Fig 2). The first group are transparent bright spheres, while others appear translucent opaque spheres. Spherules display different colors, transparent, white, ochre and black as well as colorless, and various sizes, from 15 to 60 μm. The ochre spherules are between 60 and 75 μm opaque with rough appearance, resembling globigerinas. Those with black/dark brown colors are shiny and smooth, as well as silver ones that could be metallic. Their dimensions range from 40 to 100 μm, with some smaller, between 10 and 25 μm. A second group has semi-regulatory geometric shapes, matte black and are isolated, embedded in quartz or gypsum crystals. They are mostly observed in groups of tens and apparently fused. Irregular aggregates of dark gray or black porous appearance and shiny, or opaque are present. They differ from others by having larger sizes and a porous appearance. Whole and fragmented semi-irregular spherules of gray, dark gray, with vitreous luster and other characteristic spheres are observed. They are glossy black and have conchoid fractures. These particles are molten material by the appearance of their outer surface. The smallest are not fragmented and grouped into single masses. The conchoid fractures present iridescence. Large fragments measure more than 200 μm and in some cases appear to have undergone twisting. They are found almost exclusively at the base of the horizon of black clay, mixed with lignite and quartz fragments. At the base of the dark clay there are layers up to 5 mm thick of dark brown material with patches of compacted spherules.

Tektites are observed with elliptical or spherical shapes, straight flat sides and conchoid fractures with varying sizes up to 5 mm; small ones are shinny and fractures are iridescent with some opaque. Large tektites occur at the base of the black clay with sizes decreasing with position. Clusters of matte glossy appearance show sizes up to 300 μm long.

### Fossil assemblage

Remains of marine organisms and plants (algae) are covered with whole and fragmented vitreous-looking spherules. The dark/black particles penetrate the shell of pelecypods and corals (Fig 3). Spherules reached high enough temperatures to melt the shells. The shells show spherules in the wavy structure, in some cases forming oval structures. It is common to find spherules on the shells, especially of metallic appearance and black and translucent ones.

In the lower sandstone, size of rudist specimens reaches 15 cm diameter, with some up to 40 cm long. The rudists get smaller and less abundant toward the black clay so that 0.5-1 m below, only small species ~3 cm long and 1–3 cm diameter are observed. The size of gastropods and pelecypod shells decreases with position upwards in the outcrop. The corals belong to several families and are mixed with species of turritella. The gastropods near the coral-dominated zone reach 15 mm in diameter and up to 50 mm in length and are mixed with various types of pelecypods. The coral branches at the outcrop bottom reach 20 mm in diameter, while those at the top are small, about 5 mm in diameter, suggesting that water depths were likely shallow close to the beach. The bivalves were probably alive at the time of the ejecta arrival as suggested by their closed their shells. On a beach, bivalves might have been open and fragmented by the action of the waves. At 20 cm below the black clay, sizes of fossils are distinctly smaller.

### Quartz

Quartz is mainly present as an amorphous mass and in some cases, crystals that show long flat faces including spherule fragments with black impurities. Several quartz veins 3–20 mm thick run the layer horizontally, although at some points they are interspersed. The quartz crystals show planar deformation features. Thin flat crystals show PDFs, normally in one direction with some in different directions (Fig 4). The average distance between them is between 12 and 18 μm. The separation between the lines and unidirectionality is what distinguishes quartz from plaster, in which the lines show large separations (34 μm on average) and different crisscrossed directions.

### Metallic particles

Different metallic particles are found in the black clay (Fig 5a). The central part of the black clay is marked by a fine-grained <5 μm grayish to black brownish paste. The microscopic particles show ferromagnetic and diamagnetic properties, found inside quartz crystals and other minerals.

Four different types of particles and behavior are observed. The more abundant are in quartz, gypsum and grains that respond to magnetic fields. The second type appears similar to previous ones with diamagnetic behavior. These particles, instead of moving with the magnet, jump and interact with neighboring particles. Third types are aggregates of microscopic particles apparently glued to iron sheets and/or nickel of high purity. Some of these particles show rocky structures and others have high content of rare earths and with up to 35% of neodymium and elements such as niobium in concentrations greater than 30%. The last type of particle in the clay are nuggets of small sizes <50 μm and apparently composed of a single element with branched structures and spherules from 15 to 25 μm. Branched particles can reach 0.3 mm in total length and when in the presence of magnetic fields are arranged with their longest axis, perpendicular to the magnet. Grains are found in bright silver nuggets isolated or in several branches.

Optical microscope observations do not show internal structures, so we speculate they are pure metals.

### Gypsum

The abundant gypsum crystals show various shapes and sizes ranging from 0.1 mm to more than 8 mm, with fragments of lenticular aggregates. Thickness is around 0.2 mm maximum. Scanning electron microscope, energy dispersive spectroscopy and Raman analyses show that impurity-free crystals are pure calcium sulfate. In some parts the gypsum is interspersed by anhydrite fibers. Raman spectra show gypsum lines: 415, 495, 621, 672, 1009 (main), 1141, 3405 cm.

At the base of the black clay, lenticular aggregates show tens to thousands thin sheets of gypsum in lenticular structures with approximate hexagonal bases. Their overall shape is a double hexagonal prism/cone. Each crystal is thin, less than a few µm thick and can be up to 8 mm long. The whole lenticular aggregates are almost horizontal reaching up to 8 mm in diameter and 2 mm height. Each sheet of gypsum is flat and transparent. It is common to observe small ash specks, spherules and other impurities between sheets. When observed under an optical microscope, different levels can be seen by modifying the instrument focus. Illuminating the crystals with white LED light and changing the orientation results in iridescence changes and thinner crystal birefringence. This appears randomly distributed with sometimes dark brown and black colors. In some parts they are isolated, while in others they are grouped and separated <1 mm apart and randomly oriented.

The tabular crystals are distributed in two levels, with well-preserved crystals in the lower level and fragmented ones in the upper level. The crystals are in the form of lentils and hexagonal double prisms, dark brown or black due to contamination by thin layers of microcrystals and spherule fragments. The diameter ranges from 2 mm to 8 mm, while the thickness is from 0.5 mm to 3 mm. If compressed, layers are easily cut showing the bright crystals. Crystals present PDFs with different directions (Fig 5b), Crystals appear affected by the shock wave and not all Crystals are perfectly stacked, they are observed in different planes. Among the mixed tabular crystals, there are fractured crystals with PDFs displaying torsion. Due to different structures compared to quartz and low hardness, average distances between the lines is 32–36 μm. Another difference between the PDFs in plaster is that most gypsum grains are decorated. The crystals are almost completely transparent and embedded impurities can be observed.

The tabular crystals are lenticular fragments distributed randomly in the black clay. Their dimensions are reduced as they approach the top. Each fragment is a sandwich of several sheets, sometimes they preserve a double-tipped lanceolate shape however most of the time they are just irregular fragments. Their dimensions at the base can be up to several mm in length and 0.2 mm thick. At the top of the black clay, particles are less than 1 mm long and 0.1 mm thick. Tabular crystals are thin 1–3 μm thick and stacked in only one direction. Between each crystal are abundant impurities of various minerals. The flakes present PDFs, which are unlike those present in quartz, with different directions and in different planes.

### Major and trace elements

The petrographic, SEM, EDS and chemical analyses of the black clay and upper and lower sandstones allow to characterize the section. The lower sandstone contains: C, O, Na, Mg, Al, Si, S, K, Ca, Ti, Fe and Zr. The upper sandstone contains C, O, Mg, Al, Si, S, K, Ca, Ti, Fe and unlike the one below, a small content of Ba and Zn appears, but Na and Zr have not been found. In the inner part of the black clay, it is found in addition to C, O, Na, Mg, Si, P, S, K and Ca, occurrence of Cl, Cr, Mn, Cu, Zn, As, Se, Zr, Nb, Mo, Rh, Ag, Sb, Ba, Pr, Nd, W, Os, Ir, Pb and Bi. The Al, Ti, Fe and Ni contents are higher than in the lower and upper sandstones.

**Fig 7:**
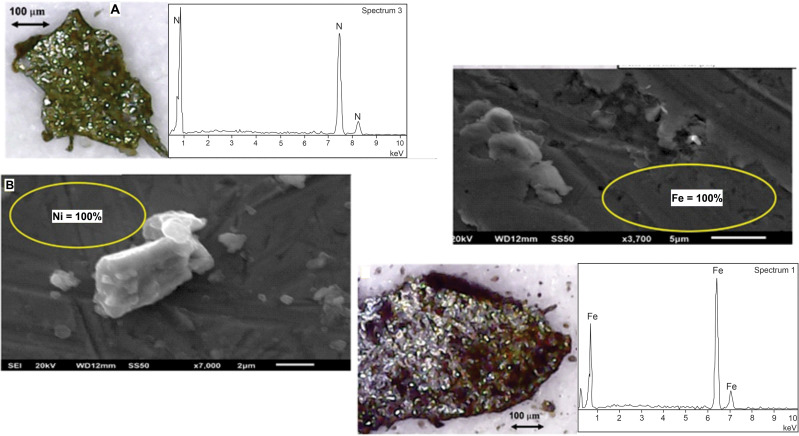
Scanning electron SEM analysis and elemental composition of impurities of pure iron and nickel. Images of the metallic particles analyzed are shown, with the electron microscopy images and corresponding SEM graphs.

**Fig 8:**
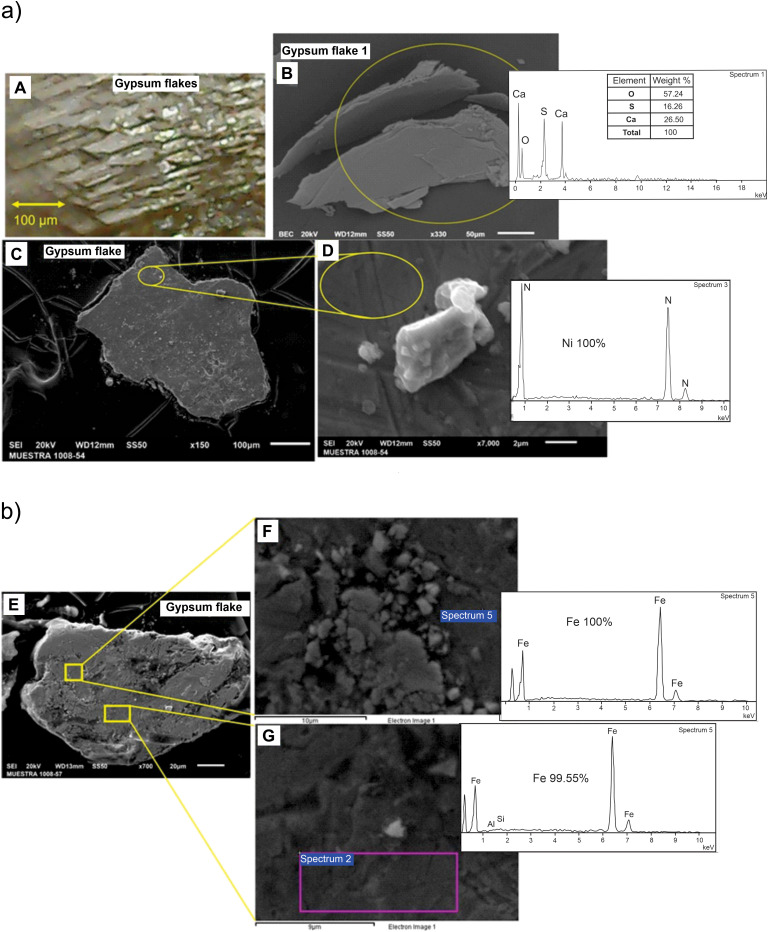
(a) Scanning electron analysis and elemental composition on gypsum flakes, with elemental analyses of iron and nickel inclusions in gypsum. (b) Scanning electron image of broken zircon.

In the black clay, analyses on the crystals document platinum group elements (Ir, Os, Pd) and Fe and Ni (Fig 6). Crystals of quartz, calcite and gypsum, with impurities and dark and translucent particles of few micron sizes, dark in color are stuck/embedded in the crystals. Analyses on crystals show Os, Ir, Nd, Pr and Pd (Fig 6). Metallic particles are made of Fe or Ni (Fig 7). Fe and Ni metals are found isolated particles, agglomerates, inclusions in other particles and in gypsum. The size ranges are less than 0.5 mm and elongated in shape. Twenty-four particles were analyzed with 255 measurements and 38 elements detected. This includes analyses of metallic particles, impurities in gypsum flakes and on fragmented zircons (Figs 7 and 8).

**Fig 9:**
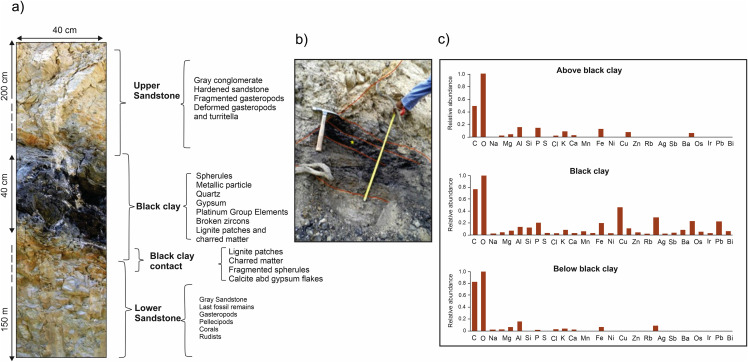
Rayon reef K/Pg boundary section, with the black clay and the lower and upper sandstones. (a) Rayon section with summary of characteristics of clay layer, lower and upper sandstone layers. (b) Close up view of clay layer. (c) Geochemical data for the clay layer and lower and upper sandstones.

The overall particle analyses indicate Fe is present in 194 points, Ni in 43 points, rare earths in 94 points, Pr in 14, Nd in 79 and Pm in 1. PGEs in 7 (Rh (1), Os (5), Ir (1). Ir, Os, Pt, Pd and Rh are detected in samples picked up at random in the black clay. Samples marked “Sx” are dark particles showing ferromagnetic response. They have sizes between 100–300 µm. samples marked “VCx” are transparent or translucid crystals that can be separated from dust by means of a magnet. These samples were collected near the center of the black clay horizon above the spherule rich layer. Major and trace element analyses for the black clay and lower and upper sandstones are summarized in Fig 9.

## Discussion

The Rayon section records the sequence of events across the gulf with the Chicxulub impact. Formation of large impact craters involves high-energy release in short time scales [43]. The impact dynamics involves extreme processes at the end of the range scale, with distinct boundary conditions, rheology, temperatures and pressures. The Chicxulub impact excavated a deep transient cavity several kilometers deep, with a high temperature blast and seismic waves travelling around the Gulf of Mexico and Caribbean Sea. The heated target rocks of fragmented ejecta formed a tall impact plume and lateral curtains, with a high-energy blast and ballistic hot droplets ejected. The K/Pg boundary at distal and intermediate locations shows a dual-layer with a basal spherulitic layer capped by a clay layer of fine-grained ejecta [22]. The cap layer formed by fine ejecta and dust that reached the top of the atmosphere and redeposited several months after the impact. Proximal sites in the gulf show a high-energy clastic unit associated with the tsunami and debris flows, overlain by the fireball layer of ejecta reentry to the atmosphere. In proximal sites the iridium anomaly lies in the fireball layer in contrast to distal sites where it is in the spherulitic layer [2].

The Rayon reef inland the platform records the events at the time of the impact. The transition zone at the base of the black clay shows deformation induced by the seismic waves before the expansive shock wave and high temperature blast. This is followed by impact ejecta of spherules, fragmented spherules, shocked quartz, crystals, metallic particles and gypsum. Low-energy ejecta around the impact site produced vegetation fires marked by abundant lignite patches. Their distribution depended on the trajectories, relative distance to impact site and location conditions. The atmospheric disturbance was intense in the circum-Gulf area, with the high-temperatures resulting from the impact and the high-temperature impact plume. In the reef, bivalve shells record the impacting spherules. The black clay basal layers are enriched in impact-derived markers, with iron and nickel particles and high REE contents further linking the mineral assemblage to the impact.

Distribution of reef organisms shows impact effects, with charred and semi-charred plant matter, zones covered by lignite, mixed with spherules and pelecypod shells damaged by spherules. The mixed fossil assemblage supports a sudden disturbance. The sizes of rudist specimens reach 15 cm diameter, with some up to 40 cm long. The bivalves get smaller and less abundant toward the black clay so that 0.5-1 m below, only small species ~3 cm long and 1–3 cm diameter are observed. The size of gastropods and pelecypod shells decreases with position in the outcrop upwards to the black clay base. The corals from various families are mixed with turritella species. The gastropods near the coral-dominated zone reach 15 mm diameters and up to 50 mm lengths, mixed with various types of pelecypods. The branches of corals, at the outcrop bottom, reach 20 mm in diameter, while those at the top are small, only ~5 mm in diameter. This suggests that the water depth was decreasing with high levels close to the beach.

The mixed occurrence of PGE, REE and carbonates from the Yucatan shelf, foraminifera, marine fossils and deep-water seafloor sediments indicate high-energy conditions. The clams and shells were close to the beach in shallow waters, with incoming spherules damaging them before cooling. Rests of marine and algae are covered with whole/fragmented vitreous-looking spherules. Dark spherules penetrated the pelecypod shells, embedded in the plant fibers.

Ejecta deposits at intermediate distances in the gulf area around 10–20 crater radii show evidence of fragmentation (comminution) of projectile and target. Part of the melted material cooled and solidified during flight. The abundant large tabular gypsum and lentil-shaped crystals could have been transported from the impact site in the form of molten droplets. Part of the carbonates and especially the calcium sulfates were fragmented but not completely melted and are scattered and mixed in the ejecta.

The impact energy generated ejection of incandescent particles with widespread vegetation fires [11,41,42]. At the Rayon site, burned remains of vegetation/fauna and semi-charred matter on the reef, as well as algae, locally or wind transported. Between the sand layers and between quartz and gypsum crystals, there are small tubular ash particles of charred matter with 10–35 μm length and 5–15 μm diameter. Some embedded inside quartz or placed almost vertically in semi-charred regions of reddish color. Particles show black colors and bright silver/gold colorations if covered by thin transparent quartz layers.

The abundance of charcoal supports the occurrence of burned vegetation from sites around the gulf, related to thermal radiation induced fires from the impact plume and reentering ejecta. At the Rayon site, charred matter, charcoal and lignite at the base of the black clay suggests burning from the thermal radiation, plus later addition from ejecta reentry and wildfires. The charred and semi-charred plant matter, lignite mixed with spherules, pelecypod shells damaged by spherules and the mixed fossil assemblage support sudden disturbance. Charcoal has also been found in sediment deposits on the peak-ring borehole column, which was associated with fires in the circum-Gulf of Mexico [19].

The shocked quartz grains with planar deformation features, abundant spherules and broken zircons indicate high pressure deformation (Figs 4, 5 and 8). The black clay includes a layer with abundant gypsum crystals rich in sulfates, which we associate with the carbonate ejected fragments from the Chicxulub target zone. Analyses on the crystals document Fe and Ni, rare earths and enrichment of PGE (Ir, Os and Pa), Nd and Pr (Figs 6 and 7). Further analyses on crystals are needed. The impurities in quartz, calcite and gypsum crystals show small sizes of a few microns, dark in color and stuck/embedded in the crystals at the surfaces.

The strata below the black clay contain abundant reef fossils, placed in cream-colored sandstones that change to gray near the contact with the black clay. From bottom to top are rudists, corals, turritella, gastropods and pelecypods. About 30 cm above is a level rich in deformed gastropods and turritella, with no other fossil remains. The upper sandstones show a few gastropods and fragmented shells. Higher up, embedded in compact cream-colored sandstones, there are gastropods of different sizes (5–35 mm long) and towards the top, the gray clay unit has gastropod and turritellas.

Formation of large impact craters involves high-energy release in short-time scales [1,2,4,14,43]. Impact on the Yucatan platform excavated a deep transient cavity, with intense deformation and seismic waves travelling around the Gulf of Mexico-Caribbean Sea. Fragmented heated target rocks were ejected forming the ejecta plume and lateral curtains, with ballistic hot droplets ejected [40]. Crater formation and post-impact processes have been reconstructed from studies on the crater structure and stratigraphy, numerical models and worldwide K/Pg boundary sections [e.g., 2,14,44–46]. In the crater, the basement, melt and breccias record crater formation stages, with high temperatures and energetic conditions, basement uplift, crustal fragmentation and deformation, ejecta plume and lateral curtains, breccia emplacement and post-impact resurge, seiches and tsunamis. Thinning-upward sorted breccias were deposited in the crater floor, followed by cross-bedded gravel sediments with polycyclic aromatic hydrocarbons and covered by charcoal fragments. On the peak-ring, basal sediments show a cross-bedded layer, recording seiches and reflected tsunami waves, from gulf disturbance [14].

In the Yucatan platform, it took time for conditions to reestablish, as recorded in breccias-carbonates contact, basal carbonate sediments and fossil record at the peak-ring, with foraminifera in the carbonate sediments [47]. Impact-induced climatic and environmental effects varied with time and location [12,14,19,48]. In the target zone, the crater basin was likely open to ocean circulation [47]. Carbonate sediments above the impact breccias show reworking and erosional effects, observed in the reworked breccias in the Yaxcopoil-1 borehole and at the carbonate-breccia contact in the Santa Elena borehole, which record the high-energy conditions with earthquakes and resurge disturbance [28,49].

The spherules and impact minerals found in the basal spherulitic layer in K/Pg sites are ejected ballistic material [2,19,22]. At the Guayal and Bochil sites in southern Mexico, spherulites reach large centimeter wide sizes as compared to millimetric-to-sub-millimetric sizes farther away [50,51]. The boundary layer at intermediate and distal sites is capped by the fine-grained ejecta and dust emplaced at the top of the atmosphere and redeposited in a period of several months after the impact. The role of anhydrites, gypsum and sulfur rich matter has been intensively studied [e.g., 52–55]. The massive release of green-house gases and sulfur-rich compounds have been linked to climatic changes and organism extinctions [1,2,52]. Recent assessment of release of sulfur-rich compounds indicates reduced contributions from the Chicxulub impact [53]. The analysis has been based on data from drilling projects carried out on the carbonate platform [29,30,56] The abundant gypsum at the Rayon site contrasts to findings in borehole cores in the target area. The boundary sections in the circum-Gulf of Mexico-Caribbean Sea show high-energy clastic units associated with the tsunamis and debris flows [5,23], which are overlain by the layer heated by ejecta reentry [11,41,42].

The impact-induced deformation and disturbance in the Gulf of Mexico-Caribbean Sea have been investigated at several sites along the coast and the interior. The tsunami deposits were recognized at the Brazos River site in southern Texas, which provided early evidence on the End Cretaceous impact in the region [3]. Subsequent studies have provided additional constraints and resolution on the impact events and chronology [57–61]. Irizarry et al. [58] reexamined the stratigraphy of the basal units over Late Cretaceous section, associating them to sediment flows following the impact. The lower unit is interpreted as a debris flow produced by seismic-induced ground shaking. The analyses indicate a locally derived unit and fossil assemblage. The overlying unit also interpreted as a cohesive debris flow is marked by high mud contents, possibly associated to the tsunami or an impact-induced local shelf collapse. The fossil assemblage was derived from shoreward settings and laterally transported across the shelf [58].

Accretionary lapilli of carbonate composition have been documented in the Brazos River and other sites, including the New Jersey drill boreholes [60]. The lapilli fragments are composed of low-Mg calcite microspars rich in sulfur with 0.05 to 0.3 cm sizes. The lapilli are associated with the carbonate crystals generated within the vapor plume, suggesting large amounts of particulate carbonates incorporated in addition to the spherules, shock quartz, tektites and impact formed minerals [60].

Studies in the Brazos River and sections in the area have documented the stratigraphy and macrofaunal fossil record. Studies have analyzed the impact deformation effects and relations with organism extinctions, including the cephalopod and ammonite records [61].

Studies at more distal sites, include the Tanis site in the Western Interior, ~ 3,050 km away, where an earthquake-triggered seiche produced a turbulent deposit with marine and continental fossil remains and impact ejecta [32]. The boundary layer of the ejecta shows spherules, microkystites, shocked minerals with multiple PDF sets, unaltered impact-melt glass and iridium anomaly. The event sequence associates the chaotic marine-fluvial fossil assemblage to effects of local deformation and ejecta arrival minutes after impact. To the south, in the Gorgonilla island section, ~ 3,050 km away, the boundary is marked by a spherule-rich layer. The ejecta layer preserves a remarkable assemblage of impact derived material. Studies of distribution, sizes, morphology, internal structures, and mineralogical and chemical compositions provide constraints on ejecta transport and distribution [31,55].

Ejecta deposits at intermediate distances around 10–20 crater radii show evidence of comminution of projectile and target. Field studies and numerical models constrain the impact ejecta global distribution [2,9,22]. Part of ejected melted material cooled during flight. The abundant large tabular gypsum and lentil-shaped crystals could have been transported from the impact site in the form of molten droplets. Part of the carbonates and calcium sulfates were fragmented but not completely melted and are scattered in the ejecta. The high-resolution models for ejecta emplacement at far distances by Artemieva and Morgan [62] constrain mechanisms for the spatial/temporal distribution and role of atmospheric interactions. Models show that ejecta traveled long distances in short times. Circum-Gulf of Mexico/Caribbean locations are affected by tsunamis and debris flows, with partial complex stratigraphic records.

Nearby sections like La Popa basin to the north provide evidence on the widespread disturbance from tsunamis and debris flows far away of coastline. Site records erosion of the shallow shelf with deposition of mixed debris and ejecta-rich dense flows [5,23]. The basal unit lies over an erosive surface showing large sandstone fragments and shallow water debris with mixed fossil remains and silicic and carbonate spherules. The unit is conformably covered by laminated sandstones recording re-establishment of sedimentary deposition. Fossil remains include, in addition to bivalve and gastropod shells and algae, mixed remains of dinosaur and mosasaur bones and teeth [5]. Unraveling event sequence records from proximal boundary sections provides constraints on ejecta emplacement and impact effects.

The location of the reef inland in the Valles-San Luis Potosi platform provided a protected location to preserve impact-induced effects. Before arrival of the tsunami waves, arriving ejecta in the black clay is marked by spherules, fragmented spherules, shocked quartz, tektites, metallic particles and gypsum. The Rayon reef record constrains ejecta arrival and impact-induced effects across the Gulf.

## Conclusions

The Rayon reef boundary section in the Valles-San Luis platform preserves a high-resolution record of the Chicxulub impact. Section shows three distinct units. The lower sandstone with a fossil assemblage of rudists, followed by corals, bivalves, turritellas and gastropods. The upper sandstone layers show a ~ 10 cm unit above the black clay contact. with no fossil remains, followed by a ~ 80 cm unit with fragmented gastropods and turritellas. The black clay, characterized by absence of fossil remains and distinctive mineralogy, shows shocked quartz with PDF deformation, spherules, metallic particles, pure iron and nickel inclusions, broken zircons and REE and PGEs that link it to the Chicxulub impact. The black clay basal layer shows gypsum and calcite crystals with lignite patches, fragmented spherules and crystals, ash and semi-charred vegetal matter covered by lignite mixed with spherules, and corals and pelecypod shells incrusted by spherules. The reef environment provided a protected location that preserved impact-induced effects, with the event sequence supporting a sudden disturbance.

## Supporting information

S1 File The supporting information pdf file includes text and two tables: S1 Text. Rayon reef, Valles-San Luis Potosi platform, S1 Table 1. Elemental data and S2 Table 2. Scanning electron chemical data.
(PDF)
