# Supplementary material for: Unraveling the Cretaceous-Paleogene boundary event across the Gulf of Mexico—High-resolution Rayon reef section, Valles-San Luis Potosi platform, Mexico
Source: PLoS One. 2026 Apr 22;21(4):e0345692. doi: 10.1371/journal.pone.0345692 (PMC13102201; doi:10.1371/journal.pone.0345692)
Supplement: S1 File — Rayon reef, Valles-San Luis Potosi platform, S1 Table 1. Elemental data and S2 Table 2. Scanning electron chemical data. (PDF) [file pone.0345692.s001.pdf]

PONE-D-25-28560R1
Suporting Information File
Dataset

Unraveling the Cretaceous-Paleogene Boundary Event Across the Gulf of Mexico – High-Resolution Rayon Reef Section, Valles-San Luis Potosi Platform, Mexico

Roberto Bartali, Jaime Urrutia-Fucugauchi, Jose Ramon Torres-Hernandez, Ligia Perez-Cruz, Rosa Lina Tovar-Tovar

Rayon reef, Valles-San Luis Potosi platform

The study area in the Valles-San Luis Potosi platform lies farther to the east and south of Cretaceous/Paleogene K/Pg boundary sections in northeastern Mexico. Those sections were in locations at the shoreline affected by the tsunamis and high energy processes. Paleoreconstructions of the Gulf of Mexico coastal area show complex abrupt relief associated to thrusting and folding during the Laramide orogeny in the Late Cretaceous and Paleogene.

The section in the Cardenas Formation is formed by clastic-carbonate sediments overlying carbonate sediments of the Valles-San Luis Potosi platform. The Cardenas Formation is formed by three members of marls, silts and sandstones, with the basal and upper members characterized by coral-rudist limestones. Studies have investigated the stratigraphy, depositional environment and fossil contents of the Cardenas Formation, as well as the structural and tectonic deformation in the Sierra Madre Oriental. In the section, the most abundant assemblage corresponds to rudists and corals. The overlying layers contain isolated small specimens of rudists (radiolitids), corals and turritellas.

Element			O	Al	Si	P	S	K	Ca	Fe	Ni	Zr	Rh	Ba	Pr	Nd	Os	Ir	Pb
Figure	Image																		
crystal 3-1	4P	weight %	37.940				8.070		11.510					39.110	0.430	2.000	0.930		
		moles	2.371				0.252		0.287					0.285	0.003	0.014	0.005		
Fig. 6a		# of atoms	777.088				82.472		94.110					93.325	1.000	4.544	1.602		
SEM horizonte	polvo 2C-8A	weight %	2.960				19.080			7.580			0.220						70.150
cristal xx3		moles	0.185				0.595			0.136			0.002						0.339
Fig. 6b		# of atoms	86.540				278.332			63.489			1.000						158.363
SEM horizonte	JRTH IG 4P3	weight %	48.070	3.400	6.130	9.050		0.870									24.420	8.050	
cristal xx3		moles	3.005	0.126	0.218	0.292		0.022									0.128	0.042	
Fig. 6c		# of atoms	135.027	5.663	9.809	13.130		1.000									5.769	1.882	
	gypsum flake	weight %	57.620				19.670		22.710										
		moles	3.601				0.613		0.567										
Fig. 8ab		# of atoms	6.356				1.083		1.000										
	Zircon	weight %	45.630	1.120	13.210				0.480	2.650		36.910							
		moles	2.852	0.042	0.470				0.012	0.047		0.405							
Fig. zircon		# of atoms	238.135	3.466	39.272				1.000	3.962		33.783							
	1008-54	weight %									100.000								
		moles									1.704								
Fig. 8ad		# of atoms									1.000								
	1008-57	weight %								100.000									
		moles								1.791									
Fig. 8bf		# of atoms								1.000									
	1008-57	weight %		0.210	0.650					99.550									
		moles		0.008	0.023					1.783									
Fig. 8bg		# of atoms		1.000	2.974					229.036									

S1 Table 1. Elemental data

Sample	Element	Weight %
Crystal 3-1	O	37.94
	S	8.07
	Ca	11.51
	Ba	39.11
	Pr	0.43
	Nd	2.00
	Os	0.93
Crystal 3-2	O	31.98
	Al	1.22
	Si	0.09
	Mn	63.01
	Fe	1.13
	Nd	0.59
	Os	1.17
JRTH IG 4P3	O	48.07
	Al	3.40
	Si	6.13
	P	9.05
	K	0.87
	Os	24.42
	Ir	8.05
Gypsum flake (metallic particle)	Ni	100.00
Gypsum flake (metallic particle)	Fe	100.00
Gypsum flake	O	57.24
	S	16.26
	Ca	26.50
Gypsum flake (metallic particle)	Ni	100.00

Sample	Element	Weight %
Gypsum flake (metallic particle)	Fe	100.00
Gypsum flake (metallic particle)	Fe	99.55
Broken zircon	O	45.53
	Al	1.12
	Si	13.21
	Ca	0.48
	Fe	2.65
	Zr	36.91
Crystal 4P-3	O	48.07
	Al	3.40
	Si	6.13
	P	9.05
	K	0.87
	Os	24.42
	Ir	8.05
Crystal 4P-3	O	45.39
	Al	32.12
	Si	1.92
	Mn	12.91
	Fe	1.34
	Nd	1.86
	Pm	0.23
	Os	4.23
Gypsum flake (metallic particle)	Ni	100.00
Gypsum flake (metallic particle)	Fe	100.00
Gypsum flakes	O	57.24
	Si	16.26
	Ca	26.50

S2 Table 2. Scanning electron chemical data
